# Supplementary material for: Low Gut Microbial Diversity Augments Estrogen-Driven Pulmonary Fibrosis in Female-Predominant Interstitial Lung Disease
Source: Cells. 2023 Feb 28;12(5):766. doi: 10.3390/cells12050766 (PMC10000459; doi:10.3390/cells12050766)
Supplement: Supplementary file 1 [file cells-12-00766-s001.zip › cells-2203908-supplementary.pdf]

Supplemental Figures

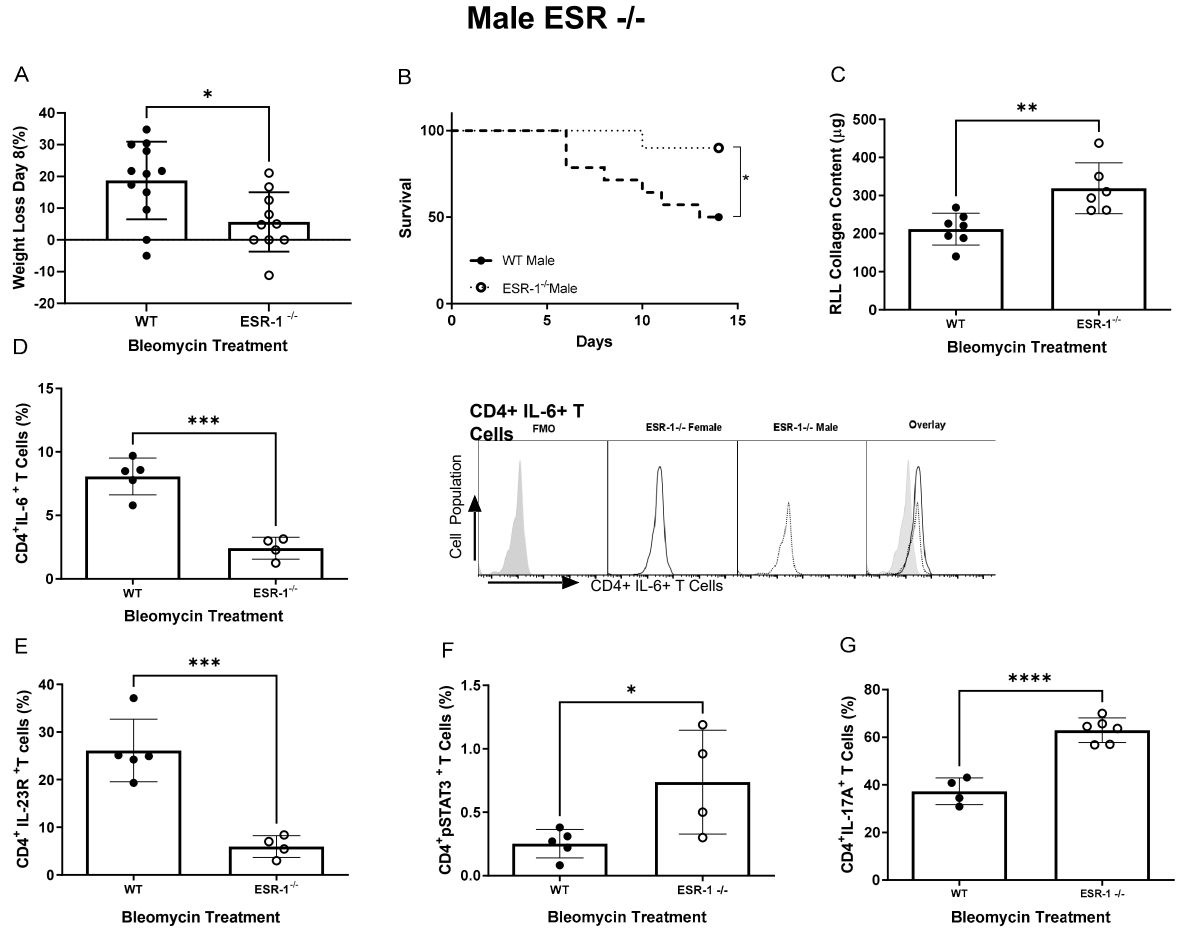

Supplemental Figure S1: Loss of estrogen receptor alpha subunit (ESR-1) improves male survival and augments fibrosis. A) WT and ESR-/- mice were treated with bleomycin and monitored for 14 days A) Body weights of mice at day 8 compared to day 0; **B)** Murine mortality across 14 days. Kaplan-Meier survival analysis with log-rank test was used to determine differences between groups; **C)** Soluble collagen content of RLL by Sircol assay. Flow cytometric analysis of T cells from single cell lung suspensions at day 14 for **D)** IL-6; **E)** IL-23R; **F)** pSTAT3Y^{705} and **G)** IL-17A. Comparisons between cohorts were performed using one-way ANOVA with Tukey's post-hoc. *P < 0.05, **P < 0.01, ***P < 0.001, ****P < 0.0001, ns: no significance. RLL: Right lower lobe. Bars are mean ± SD; each symbol represents an individual mouse. WT: Wildtype, ESR-1-/-: estrogen receptor alpha knockout mice.

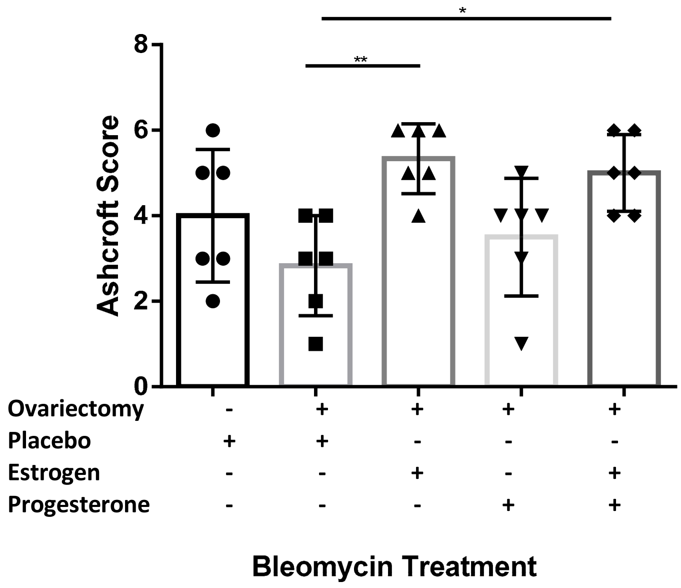

Supplemental Figure S2. Ovariectomized mice display decreased lung fibrosis. Ashcroft scoring of sham and ovariectomized mice treated with placebo, 17β-E2, progesterone, or both. Comparisons between cohorts were performed using student's one-way ANOVA *P < 0.05, **P< 0.01

Supplemental Tables

Table S1. Mouse strains used in this study.

Mouse Strain	Description	Designated Name	Vendor
Wild-type (WT) C57BL/6J	C57BL/6 is the most widely used inbred strain. It is commonly used as a general purpose strain and background strain for the generation of congenics carrying both spontaneous and induced mutations.	WT	Jackson Laboratory (Stock No: 000664)
Estrogen receptor alpha knock out mice (ERα KO mice)	B6DN (cg) – Esr 1<tm4.2ksk>/J These mice carry a knock out mutation of the Esr1 gene in which exon 3 has been deleted. Homozygotes are viable but not fertile.	$Esr\text{-}1^{-/-}$	Jackson Laboratory (Stock No: 026176)
Wild-type (WT) C57BL/6J Sham operated mice	Sham Surgery (faked surgical intervention that omits the ovariectomy)	WT Sham	Jackson Laboratory (Stock No: 000664)
Wild-type (WT) C57BL/6J Ovariectomized mice	Ovariectomy (Female mice had their ovaries surgically excised).	OVX	Jackson Laboratory (Stock No: 000664)

Table S2. Flow cytometric antibodies used in this study.

Antibody	Vendor, Catalog No	Host organism; antibody type	Clone	Conc (µg)/volume (µL) per test*
**Antibodies against human proteins**				
CD3 Alexa Fluor 700	BD 557943	Mouse Monoclonal	UCHT1	1.25 µl
CD3 BV785	Biolegend 317330	Mouse Monoclonal	OKT-3	5 µl
IL-6 APC	BD 561441	Rat Monoclonal	MQ2-13A5	5 µl
IL-17A PE	Biolegend 512306	Mouse Monoclonal	BL168	5 µl
STAT3 (pY705) Pacific blue	BD 560312	Mouse Monoclonal	4/P-STAT3	10 µl
Lap TGF-β1 PerCP/Cy 5.5	Biolegend349612	Mouse Monoclonal	TW4-2F8	1.6 µl
Lap TGF-β1 APC	Biolegend 349706	Mouse Monoclonal	TGW4-6H10	1.6 µl
RORC AF488	BD 563621	Mouse Monoclonal	Q21-559	2.5 µl
**Antibodies against murine proteins**				
CD3 Alexa Fluor 700	Biolegend 100216	Rat Monoclonal	17A2	0.25 µg
CD4 APC-Cy7	Biolegend 100414	Mouse Monoclonal	GK1.5	0.1 µg
IL-17A PE	Biolegend 506904	Rat Monoclonal	TCII-18H10.1	0.1 µg
IL-17A BV 650	Biolegend 506930	Rat Monoclonal	TCII-18H10.1	1.6 µg
STAT3 (pY703) Pacific Blue	BD 560312	Mouse Monoclonal	4/P-STAT3	10 µl
IL-6 FITC	Invitrogen 11-7061-82	Rat Monoclonal	MP5-20F3	1.6 µg
IL-6 APC	Biolegend 504808	Rat Monoclonal	MP5-20F3	0.04 µg
IL-23R APC	Biolegend 150906	Rat Monoclonal	12B2B64	0.02 µg
GP-130 PE	Biolegend 149404	Rat Monoclonal	4H1B35	0.02 µg
TGF-β1 PerCP/Cy5.5	Biolegend 141410	Mouse Monoclonal	TW7-16B4	0.13 µg
LIVE DEAD	Invitrogen L34967			1:1000

*Test = 1 million cells in 50µl of staining buffer.

Table S3. Genomic regions demonstrating ERα binding in T-cells in the locus of *STAT3*. Chromosome positions are given in hg38 coordinates. Statistics are calculated by MACS2.

Chromosome	Start	End	Peak length	-log10(p-value)	Fold enrichment over input	-log10(q-value)
chr17	42312484	42313455	972	4.66916	2.72445	1.90148
chr17	42314875	42316383	1509	4.17741	2.58679	1.49223
chr17	42337598	42338670	1073	4.51024	2.68161	1.76113
chr17	42363913	42364381	469	3.57088	2.40576	1.01901
chr17	42377801	42378844	1044	3.84965	2.41173	1.25401
chr17	42387418	42389449	2032	4.75049	2.71867	1.98772

Table S4. Sample demographics of ChIP-seq donors and resulting data. Remaining reads count the number of trimmed, decontaminated reads following FASTQ preprocessing. The percentage of properly paired, aligned reads is given, as is the number of peaks identified by MACS2 for each sample relative to the input sample.

	Sex	Age	Input Reads	Remaining Reads	% Properly aligned reads	# Enriched Peaks
**Input**	n/a	n/a	156713964	156492276	99.27	n/a
**p1035862-9**	F	22	140187522	139974386	99.38	2412
**p1035907-9**	F	68	135521910	135246434	99.20	1012
**p1035916-5**	F	30	136026564	135808588	99.16	2181
**p1035928-8**	F	71	128434534	128086940	99.13	15210
**p1035941-1**	M	24	122478960	122245832	99.41	2866
**p1035953-2**	M	32	132110454	131866146	99.05	2756

Table S5. Significantly enriched functional categories for genes associated with ERα binding regions by GREAT. Significantly enriched categories met a binomial FDR Q-value threshold of 5%.

Ontology	# Term Name	Binom Rank	Binom FDR Q-Val
GO Biological Process	DNA replication-dependent nucleosome assembly	45	3.41115e-14
	protein refolding	69	5.99735e-11
	positive T cell selection	72	1.88179e-10
	positive regulation of nuclear-transcribed mRNA catabolic process, deadenylation-dependent decay	79	3.96028e-10
	positive regulation of mRNA catabolic process	80	4.38968e-10
	T-helper cell differentiation	108	4.94657e-8
	positive regulation of natural killer cell activation	113	1.59572e-7
	natural killer cell differentiation	117	2.36310e-7
	chromatin silencing at rDNA	120	2.89391e-7
	T-helper 17 cell differentiation	168	1.13721e-5
	CD4-positive or CD8-positive, alpha-beta T cell lineage commitment	197	6.09469e-5
	telomere capping	209	7.80781e-5
	spliceosomal tri-snRNP complex assembly	263	3.67336e-4
	stress granule assembly	296	8.86802e-4
	ribosomal small subunit assembly	355	3.47249e-3
Cellular Component	nucleosome	4	1.17346e-15
	DNA packaging complex	8	4.23471e-13
	MHC protein complex	9	8.12117e-13
	integral component of lumenal side of endoplasmic reticulum membrane	21	5.32903e-6
	alpha-beta T cell receptor complex	22	1.01614e-5
	MHC class II protein complex	25	6.36248e-5
	PTW/PP1 phosphatase complex	45	3.91793e-3
	MHC class I peptide loading complex	49	7.12120e-3
	PRC1 complex	53	1.44796e-2
GO Molecular Function	peptide antigen binding	9	8.11280e-9
	MHC class II protein complex binding	26	5.08035e-5
	snRNA binding	43	2.78861e-4
Mouse Phenotype Single KO	abnormal memory T cell physiology	23	7.41443e-16
	decreased B-1a cell number	45	1.99471e-10
	abnormal thymocyte activation	50	7.40996e-10
	decreased activated T cell number	98	7.31205e-6
	decreased level of surface class I molecules	100	1.08094e-5
	abnormal level of surface class I molecules	107	2.37547e-5
	absent CD8-positive, alpha-beta T cells	116	3.52385e-5
	abnormal antigen presentation via MHC class I	153	2.74616e-4
	abnormal brachial lymph node morphology	184	1.13170e-3
Mouse Phenotype	abnormal memory T cell physiology	11	3.96990e-23
	abnormal T cell selection	17	2.49581e-15

	abnormal mature gamma-delta T cell morphology	19	1.55604e-14
	abnormal positive T cell selection	23	4.89923e-14
	decreased DN1 thymic pro-T cell number	33	5.64557e-12
	abnormal thymocyte activation	46	6.33874e-10
	abnormal gamma-delta intraepithelial T cell morphology	48	1.27486e-9
	decreased spleen germinal center size	49	1.28510e-9
	decreased level of surface class I molecules	82	6.58477e-7
	abnormal level of surface class I molecules	88	1.84828e-6
	absent B-1a cells	89	2.44495e-6
	absent B-1 B cells	92	3.72019e-6
	abnormal antigen presentation via MHC class I	107	3.32432e-5
	decreased lymphoma incidence	115	4.65799e-5
	abnormal IgG2c level	184	1.21934e-3
	decreased activated T cell number	244	9.42092e-3
